# Development of a Visual Assay for Detection of Viable *Cronobacter sakazakii* Using RT-PSR and Hydroxynaphthol Blue Indicator

**DOI:** 10.3390/biology14040383

**Published:** 2025-04-07

**Authors:** Peng Wang, Qiming Chen, Yikai Wang, Xueting Sun, Zhanmin Liu

**Affiliations:** 1 School of Life Sciences, Shanghai University, No. 99 Shangda Road, Shanghai 200444, China; w3210407903@shu.edu.cn (P.W.); cqm2016@shu.edu.cn (Q.C.); ykaaai1998@shu.edu.cn (Y.W.); 2 Nantong Customs of the People’s Republic of China, No. 102 Chongchuan Road, Nantong 226006, China; sunxtjnu@163.com

**Keywords:** *Cronobacter sakazakii*, visual detection, reverse transcription-polymerase spiral reaction, hydroxynaphthol blue

## Abstract

*Cronobacter sakazakii* is a foodborne pathogen in powdered infant formula, which poses a significant risk to susceptible populations such as infants and the elderly. This study aims to develop a visual detection method for viable *C. sakazakii* using the reverse transcription-polymerase spiral reaction and hydroxynaphthol blue indicator. Under the optimized conditions, the detection process could be completed within 55 min with low equipment dependence. It was evaluated to have high specificity and sensitivity with the detection limit low to 1.2 × 10^1^ CFU/mL. The assay also showed 100% accuracy in artificially contaminated samples.

## Simple Summary

*Cronobacter sakazakii* is a hazardous foodborne pathogen frequently detected in powdered infant formula, posing significant health risks to neonates and immunocompromised individuals. To address the demand for rapid on-site detection, this study developed a reverse transcription-polymerase spiral reaction assay coupled with hydroxynaphthol blue for real-time monitoring of viable *C. sakazakii* cells. Under optimized isothermal conditions (42 °C), the assay could detect *C. sakazakii* cells as low as 1.2 × 10^1^ CFU/mL within 55 min. Besides, the assay could be applied in the detection of powdered infant formula samples. The assay’s low equipment requirements, rapid turnaround time, high sensitivity, and user-friendly operation provide an effective tool for food safety surveillance, clinical diagnostics, and routine monitoring in food production facilities.

## 1. Introduction

*Cronobacter sakazakii*, a kind of foodborne opportunistic pathogen in powdered infant formula (PIF) [1,2,3], which is intended to meet the sole nutritional needs of infants up to 6 months of age when breastfeeding is not possible [4]. PIF that contains *C. sakazakii* may infect infants and cause diseases like necrotizing enterocolitis (NEC), septicemia, life-threatening meningitis and even death [2,5,6,7,8,9]. Therefore, in 2002, the International Commission on Microbiological Specifications for Foods (ICMSF) also described *C. sakazakii* as a “severe hazard for restricted populations, life-threatening or substantial chronic sequelae of long duration” [10]. 

At present, there are three main types of methods for detecting *C. sakazakii*: traditional isolation and identification, immunological detection, and molecular biology detection technologies [11]. The traditional isolation and identification method is to identify the biochemical and morphological characteristics of *C. sakazakii*. It is cumbersome and time-consuming, results in low detection efficiency and is prone to false positive and false negative results [12,13]. Immunological detection is an enzyme-linked immunoassay technology based on the specific binding of antibodies and antigens, such as Enzyme-Linked Immunosorbent Assay (ELISA) [11,14,15]. Although ELISA demonstrates high specificity for macromolecular antigens, this method typically requires 4–6 h for completion and exhibits lower sensitivity (detection limit~10^4^ CFU/mL) compared to molecular techniques like polymerase chain reaction (PCR) (detection limit~10^1^ CFU/mL) [14]. Furthermore, it cannot differentiate viable from non-viable bacterial cells.

Molecular biology detection based on PCR has become quite mature for detecting pathogens such as fluorescence quantitative PCR (q-PCR) [13,16]. It has been previously reported that reverse transcription PCR (RT-PCR) can be applied to the detection of viable *C. sakazakii* [17,18]. It is worth mentioning that scientists have recently introduced a special substance to detect viable *C. sakazakii* on the basis of conventional PCR, such as propidium monoazide (PMA), ethidium monoazide (EMA) [19], and G-quadruplex DNAzyme [20]. Liang et al. introduced the sodium deoxycholate-propidium monoazide-multiplex real-time-PCR (SD-PMA-mRT-PCR) technique, which required 2 h to detect viable *C. sakazakii*, with a LOD of 10^2^ CFU/mL [21]. Lv et al. utilized immunomagnetic separation in combination with PMAxx-droplet digital PCR (IMS-PMAxx-ddPCR) and achieved a lower LOD of 23 CFU/mL [22]. In a different approach, Yu et al. developed a method combining multiplex PCR with PMA, necessitating only 80 min for viable *C. sakazakii* detection [23]. In addition, an RT-PCR triggering of a G-quadruplex DNAzyme assay for *C. sakazakki* achieved a LOD of 5.01 × 10^2^ CFU/mL using the naked eye [20]. These PCR-based assays are effective, but their expensive equipment and high laboratory requirements limit their further application. Isothermal amplification can efficiently amplify the target DNA without the requirement of expensive temperature cycling and it was widely used in the detection of viable *C. sakazakii*. Hu et al. developed an accurate method that rapidly detects viable *C. sakazakii* by combining propidium bromide with quantitative loop-mediated isothermal amplification (PMA-qLAMP) [24]. Liu et al. developed a detection method for viable *C. sakazakii* using nucleic acid aptamers based on rolling ring amplification (RCA) [25].

Polymerase spiral reaction (PSR) is a novel isothermal nucleic acid amplification method with a simple reaction system. Only one pair of primers and *Bst* DNA polymerase are required for PSR reaction, which makes the primer design and cost simpler and lower than other isothermal amplification reactions, respectively [26]. Hydroxynaphthol blue (HNB) is a kind of metal ion indicator. Its color changes with the concentration of the metal ion [27]. In the PSR amplification, the by-product pyrophosphate ion generates and reacts with Mg^2+^ in the reaction system and the dissociative Mg^2+^ concentration gradually decreases. Therefore, HNB can be used to indicate the PSR reaction process and develop a visual detection assay [28].

Therefore, in this study, a rapid visual assay for the detection of viable *C. sakazakii* was developed based on RT-PSR. The reaction temperature, reaction time and dNTP concentration of the assay were optimized. In addition, the specificity and sensitivity of the assay as well as reliability in artificially contaminated samples were also evaluated.

## 2. Materials and Methods

### 2.1. Chemicals and Reagents

DNA Marker, 6 × DNA loading buffer, Taq DNA polymerase, dNTP mixture (10 mM), RNAprep Pure Bacteria Kit were purchased from TIANGEN (Beijing, China). AMV Reverse transcriptase, *Bst* DNA polymerase, large fragment and MgSO_4_ were purchased from New England Biolabs (Beijing, China). HNB was purchased from Sigma Aldrich (Shanghai, China). PIF samples (Jinlingguan Sainamu) without contamination were obtained from Inner Mongolia Yili Industrial Group (Inner Mongolia, China). All other conventional biochemical reagents were analytically pure and purchased from Sinopharm Chemical Reagent (Shanghai, China). The experimental water was ultra-pure water with a conductivity of 18.2 MΩ. Primers were synthesized by Sangon Biotech (Shanghai, China).

Bacteria strains including *Cronobacter sakazakii* CICC 21560, *Bacillus subtilis* isolate, *Escherichia coli* ATCC 35218, *Staphylococcus aureus* isolate, *Listeria monocytogenes* isolate, *Shigella dysenteriae* isolate were preserved in the Food Microbiology and Biotechnology Laboratory, Shanghai University. *C. sakazakii*, *B. subtilis*, *E. coli*, *S. aureus*, *L. monocytogenes* and *S. dysenteriae* were cultured in ordinary Luria-Bertani (LB) solid medium, and single colonies were picked and transferred to LB liquid medium at 37 °C for 12 h.

### 2.2. RNA Extraction of C. sakazakii

*C. sakazakii* was inoculated into LB medium and incubated overnight at 37 °C with constant shaking at 150 rpm. According to the product manual of RNAprep Pure Bacteria Kit, total RNA of *C. sakazakii* was extracted from the overnight culture, and it was suspended in 100 μL elution buffer and stored at −80 °C for following use.

### 2.3. Primers of RT-PSR Reaction

The primers of RT-PSR were designed to contain an unrelated exogenous sequence (20–22 nt) at the 5′ end, while the remaining 20–22 base pairs correspond to the target region at the 3′ end [26]. To design specific primers for *C. sakazakii*, α-1,6-glucosidase gene (*gluA*) sequence of *C. sakazakii* strain Sh41g (Supplementary Material) was compared with other multiple *C. sakazakii* strains by NCBI-BLAST. Based on the specific region, four PSR primers using Primer Premier 5.0 software were designed. Among them, PSR-LF and PSR-LB were the main primers for triggering the PSR reaction, and PSR-IF and PSR-IB were the accelerating primers for increasing the reaction rate and further shortening the time for PSR amplification. The primer sequences were shown in Table 1. The G-quadruplex sequence was added to the 5′ of the PSR-LF and PSR-LB primers as an exogenous sequence. By performing both *Bst* DNA polymerase extension at the 3′ end and strand substitution at the 5′ end, RT-PSR products with repetitive target sequences were generated showing multiple banding patterns on gel [29].

### 2.4. Visual Inspection of C. sakazakii

A PSR reaction system of 25 μL was configured, and the concentrations of each component of the reaction mixture were: 2.5 μL 10 × ThermoPol buffer solution (New England Biolabs, Beijing, China), 1.6 μM PSR-LF, 1.6 μM PSR-LB, 0.8 μM PSR-IF, 0.8 μM PSR-IB, 1 M betaine, 1.4 mM dNTP, 8 U *Bst* DNA polymerase, 8 U AMV Reverse Transcriptase, 150 μM HNB and 2 μL RNA template.

The mixture was placed in a thermocycler and the program was 42 °C for 15 min (reverse transcription reaction) and 65 °C for 60 min (PSR reaction). Double-distilled water served as a blank control. The products were detected by 2% agarose gel electrophoresis at 90 V for 30 min, and the results were recorded using a gel imaging system. Besides, the color of the reaction system changed from purple to sky blue. The negative control remained purple, and the EP tubes were observed with the naked eye and photographed under natural light.

### 2.5. Optimization of Experimental Conditions

Reaction temperature optimization: RT-PSR reactions were performed at 60, 61, 62, 63, 64, and 65 °C for 60 min according to the reaction system described in “Visual inspection of *C. sakazakii*” section, respectively, followed by heating at 80 °C for 5 min to terminate the reaction. Amplification products were analyzed using 2% agarose gel electrophoresis and a gel imaging system and the visual results of color changes were recorded.

Reaction time optimization: RT-PSR reactions were performed at optimized temperatures for 20, 30, 40, 50, 60 and 70 min according to the reaction system described in “Visual inspection of *C. sakazakii*” section, respectively, followed by heating at 80 °C for 5 min to terminate the reaction. Amplification products were analyzed using 2% agarose gel electrophoresis and a gel imaging system and the visual results of color changes were recorded.

dNTP concentration optimization: RT-PSR reactions contained dNTP with final concentrations of 0.2, 0.4, 0.6, 0.8, 1.0, 1.2 and 1.4 mM were performed at optimized temperatures and times, respectively. Amplification products were analyzed using 2% agarose gel electrophoresis and a gel imaging system and the visual results of color changes were recorded.

### 2.6. Specificity of RT-PSR Visual Assay

To verify the specificity of this visual assay, five negative control strains including *B. subtilis*, *E. coli*, *S. aureus*, *L. monocytogenes*, and *S. dysenteriae* were detected. All strains (including controls) underwent identical nucleic acid extraction using the RNAprep Pure Bacteria Kit (TIANGEN, Beijing, China) following the manufacturer’s protocol. *C. sakazakii* and ddH_2_O served as positive and blank control, respectively.

### 2.7. Sensitivity of RT-PSR Visual Assay

*C. sakazakii* was cultured and its cell concentration was determined by plate colony counting. Then, the *C. sakazakii* culture was 10-fold diluted with sterile water and the sensitivity of this visual assay was evaluated by the assay developed in this study.

### 2.8. Accuracy Evaluation Using Artificially Contaminated Samples

10 g powder infant formula samples were added to 90 mL of LB liquid medium containing *C. sakazakii* with a final concentration of 10^5^ CFU/mL of viable *C. sakazakii*, and then incubated at 37 °C for 7 h. Artificially contaminated samples contained 10 g powder infant formula and 90 mL of LB liquid medium with 10^5^ CFU/mL of inactivated *C. sakazakii* (autoclaved at 121 °C for 15 min) served as negative control. Sterile water served as blank control. Artificially contaminated samples were prepared in three independent groups (viable *C. sakazakii*, nonviable *C. sakazakii*, and blank controls; n = 10 per group) and their RNA was isolated by RNAprep Pure Bacteria Kit. Each group was tested in 10 replicates, generating 30 data points in total. These experiments were designed to evaluate the accuracy of the assay developed in this study.

### 2.9. Experimental Design and Statistical Analysis

The artificially contaminated samples detection was repeated ten times and the data of artificial contamination sample testing were analyzed by IBM SPSS statistics V27.

## 3. Results and Discussion

### 3.1. Principle of RT-PSR-Based Visual Detection

**Scheme 1:**
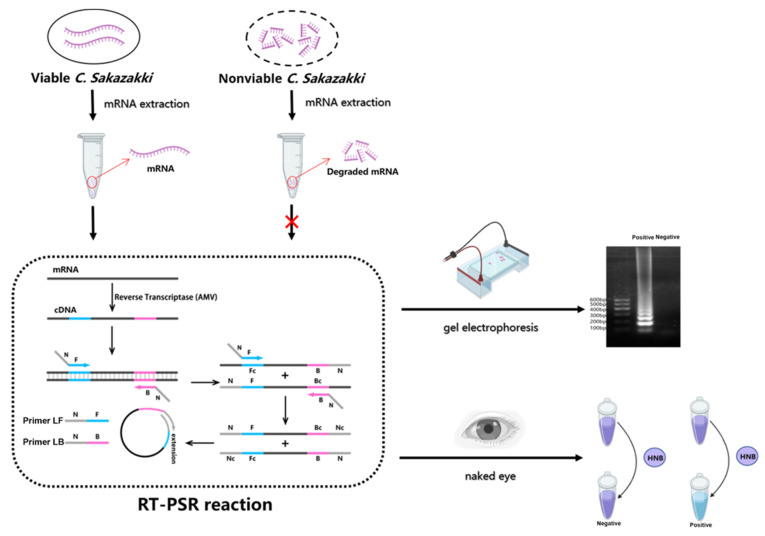
Schematic illustration for visual detection of viable *C. sakazakii* based on RT-PSR and the detail was described in Section 3.1.

The principle for visual detection of viable *C. sakazakii* based on RT-PSR was as follows:

When the *C. sakazakii* cells were viable, mRNA was undegraded and extracted. The mRNA was reverse-transcribed into cDNA using random hexamers and AMV reverse transcriptase at 42 °C for 15 min, followed by target amplification via PSR at 65 °C for 60 min. When the *C. sakazakii* cells were nonviable, mRNA was degraded, and it could not be reverse transcribed as cDNA. The PSR reaction could not be performed. During amplification, free Mg^2+^ reacted with pyrophosphate and yielded magnesium pyrophosphate precipitate. As the Mg^2+^ concentration decreased, the color of the HNB shifted from purple to blue, facilitating the visual detection of viable *C. sakazakii*. The schematic illustration of RT-PSR-based visual assay was shown in Scheme 1.

### 3.2. Optimization of Reaction Temperature, Reaction Time and dNTP Concentration for RT-PSR Visual Assay

**Figure 1:**
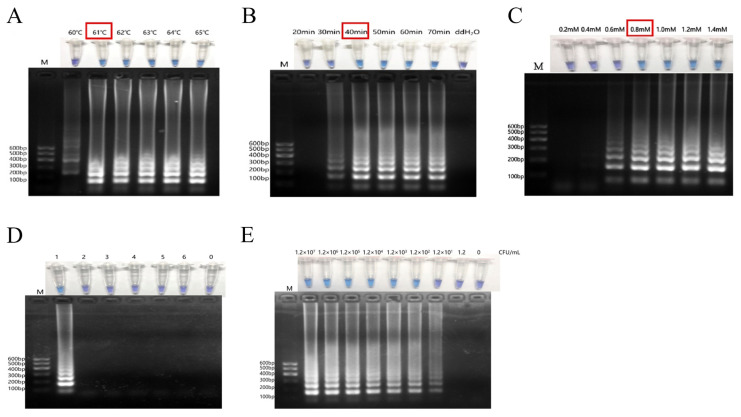
Optimization of reaction conditions, specificity and sensitivity of RT-PSR-based visual assay in this study. (**A**) Optimization of reaction temperature. (**B**) Optimization of reaction time. (**C**) Optimization of dNTP concentration. (**D**) Specificity of RT-PSR-based visual assay in this study. 1 was *C. sakazakii*, 2–5 are *B. subtilis*, *E. coli*, *S. aureus*, *L. monocytogenes*, *S. dysenteriae*, respectively, and 0 was blank control (ddH_2_O). (**E**) Sensitivity of RT-PSR-based visual assay in this study. M represented the DNA Marker. In each image, the upper half represented the visual assay, and the lower half represented the agarose gel electrophoresis. Red boxes indicate optimal reaction conditions.

The results of reaction temperature optimization are shown in Figure 1A. In the electrophoresis result, no significant gradient ladder bands were observed between 61 °C and 65 °C, while weak ladder bands appeared at 60 °C. In the visual results, all the reaction systems except the one with a reaction temperature of 60 °C showed a color change from purple to blue. This might be because there were fewer products when the reaction temperature was 60 °C, the Mg^2+^ concentration did not decrease significantly, which did not cause the HNB color change. Therefore, 61 °C was selected as the optimal reaction temperature for the RT-PSR visual assay.

For the reaction time, there was no amplification product and the color remained purple when the reaction time was 20 min. When the reaction time was at 40, 50, 60, and 70 min, there were obvious bands, and the bands became brighter with the increase in reaction time while the color of the reaction system changed from purple to blue. At 30 min of reaction, there was a faint band, but the color of the reaction system remained purple, indicating that the amount of amplification products was still relatively low. Therefore, the optimal reaction time for visualizing *C. sakazakii* by RT-PSR was determined as 40 min. The result of reaction time optimization was shown in Figure 1B.

The dNTP concentration was optimized in the range of 0.2–1.4 mM at a reaction temperature of 61 °C and a reaction time of 40 min, and the results are shown in Figure 1C. There were no DNA bands when the dNTP concentration was below 0.4 mM. The DNA bands appeared and deepened with the increase of the dNTP concentration in the range of 0.4–1.0 mM. When the dNTP concentration was in the range of 1.0–1.4 mM, the bands’ brightness didn’t change. The visual results are shown in Figure 1C (upper part). In the range of 0.2–0.6 mM dNTP concentration, the color of the product did not change to blue and remained purple. In the range of 0.8–1.4 mM, the product color changed from purple to blue. Although there were faint electrophoretic bands when the dNTP concentration was 0.6 mM, the color did not change, indicating that the amount of product was not high enough to decrease the Mg^2+^ concentration enough to change the HNB color from purple to blue. Therefore, the optimal dNTP concentration was 0.8 mM.

### 3.3. Evaluation of RT-PSR-Based Visual Assay Specificity

Extract RNA of *C. sakazakii* and other negative controls including *B. subtilis*, *E. coli*, *S. aureus*, *L. monocytogenes* and *S. dysenteriae*, were detected by the RT-PSR-based visual assay to determine the specificity. As shown in Figure 1D, only the positive control (*C. sakazakii*) could initiate the amplification and had bright bands on gel (Figure 1D, lane 1), and there was also a color change in the reaction system (Figure 1D, tube 1). The negative controls, as well as the blank control, didn’t initiate the amplification, and there were no bands on the gel (Figure 1D, lanes 2–6). There was no change in color (Figure 1D, tubes 2–6). The results showed that the RT-PSR-based visual assay developed in this study was highly specific *C. sakazakii*.

### 3.4. Determination of RT-PSR-Based Visual Assay Sensitivity

To determine the sensitivity of the visual assay developed in this study, *C. sakazakii* cell culture with known cell concentration was 10-fold gradient diluted and the RNA was extracted as the template for detection. The results showed that the lower the concentration of *C. sakazakii*, the weaker the band brightness (Figure 1E). When the concentration of the bacterial solution was 1.2 × 10^1^ CFU/mL, there was a DNA ladder band on the gel, but the color of the reaction system didn’t change until the cell concentration increased to 1.2 × 10^2^ CFU/mL (Figure 1E). Therefore, the detection limit of this visual assay was 1.2 × 10^2^ CFU/mL for the naked eye and 1.2 × 10^1^ CFU/mL for gel electrophoresis, respectively.

Compared with previous assays for detection of viable *C. sakazakii* (Table 2), the RT-PSR-based visual assay offered significant advantages. The detection time of this method was 55 min, which was greatly less than Genome-Editing Microfluidic Array coupled with digital quantitative PCR (gEMA-DqPCR) (9 h) [19], SD-PMA-mRT-PCR (2 h) [21], and One-Step RT-PCR (2.5 h) [17]. Moreover, the RT-PSR assay showcased a lower limit of detection (LOD) of 1.2 × 10^1^ CFU/mL (via gel electrophoresis) and 1.2 × 10^2^ CFU/mL (via the naked eye). This surpassed alternative assays, such as the PMA-qLAMP (4.3 × 10^2^ CFU/mL) [24] and RT-PCR employing a G-quadruplex DNAzyme trigger (5.01 × 10^2^ CFU/mL) [20]. In addition, depending on the method of isothermal amplification and visualization of the color change, it was less instrument-dependent, requiring only a conventional water bath for the assay. Notably, this assay’s distinctive capacity for immediate qualitative analysis with the naked eye distinguished it.

### 3.5. Accuracy Evaluation Using Artificially Contaminated Samples

Thirty artificially contaminated PIF samples were detected by the visual assay developed in this study. The detection results were consistent with the standard method. These indicated that the method had a 100% accuracy for artificially contaminated samples (Table 3), and there were no false positive and false negative results.

## 4. Conclusions

In this study, an RT-PSR-based visual assay for the detection of viable *Cronobacter sakazakii* cells was developed. The assay can be completed in 55 min and the results can be seen by the naked eye without the need for complicated handling or expensive equipment. In addition, the detection limits of gel electrophoresis and visual inspection are as low as 1.2 × 10^1^ CFU/mL and 1.2 × 10^2^ CFU/mL, respectively. Importantly, the method demonstrated high reliability in artificially contaminated PIF samples—a critical validation target as PIF remains the primary source of neonatal *Cronobacter sakazakii* infections. However, validation in complex matrices (e.g., dairy products, ready-to-eat foods) or clinical specimens (e.g., blood, stool) requires further investigation. Additionally, the 7-h pre-enrichment step may limit immediate diagnosis in point-of-care settings. Future work will expand validation to non-formula matrices (e.g., leafy greens, environmental swabs) and optimize clinical usability through smartphone-based colorimetric analysis. Inspired by enzymatic signal enhancement strategies [31], incorporating exonuclease III to increase visual discrimination of colorimetric results. This assay holds significant potential for controlling *Cronobacter sakazakii* contamination in both food safety and clinical applications.

## Figures and Tables

**Table 1:** Oligonucleotide primers sequences.

Primer	Sequence (5′–3′)
PSR-LF	CCCAACCCGCCCTACCCAAAGAGGAGATCCACGCGATGAT
PSR-LB	CCCAACCCGCCCTACCCAAATCGTTCATCTGGCGTAGCA
PSR-IF	AAACCGCCGATGCCTTTATC
PSR-IB	GATCATGGCGAACGGCAAG

The underlined sequences are exogenous sequences.

**Table 2:** Comparison of previous methods and RT-PSR-based visual assay in this study.

Assay	RT Time	Total Time	LOD	Reference
Aptamer combined rolling circle amplification	N/A	180 min	2.7 × 10^2^ CFU/mL	[25]
gEMA-DqPCR	N/A	540 min	10^1^ CFU/mL	[19]
PMA-qLAMP assay	N/A	40 min	4.3 × 10^2^ CFU/mL	[24]
Multiplex PCR coupled with propidium monoazide	N/A	80 min	9.5 × 10^4^ CFU/mL	[23]
SD-PMA-mRT-PCR	75 min	120 min	10^2^ CFU/mL	[21]
RT-PCR triggering of a G-quadruplex DNAzyme	90.8 min	120 min	5.01 × 10^2^ CFU/mL	[20]
IMS-PMAxx-ddPCR	N/A	110 min	23 CFU/mL	[22]
One-Step RT-PCR	132.5 min	150 min	10^4^ CFU/mL	[17]
RT-PSR-based visual assay	75 min	130 min	1.2 × 10^1^ CFU/mL (gel electrophoresis), 1.2 × 10^2^ CFU/mL (naked eye)	This work

**Table 3:** Artificially contaminated samples detection.

Samples ^a^	Visual Inspection (Positive/Total)	Culture-Based Method ^b^ Detection (Positive/Total)	Accuracy (%) ^c^
Viable *C. sakazakii*	10/10	10/10	100
Nonviable *C. sakazakii*	0/10	0/10	100
Blank control	0/10	0/10	100

^a^ A total of 30 artificially contaminated samples were prepared for this experiment, 10 samples containing viable *C. sakazakii* and 10 samples containing nonviable *C. sakazakii* 10 blank control samples. ^b^ The culture-based method refers to ISO 22964:2017 standard [30] enrichment and plating procedures. ^c^ Accuracy was calculated as (concordant results/total samples) × 100%.

## Data Availability

The original contributions presented in this study are included in the article. Further inquiries can be directed to the corresponding author.

## Supplementary Materials

The following supporting information can be downloaded at: https://www.mdpi.com/article/10.3390/biology14040383/s1, *Cronobacter sakazakii* strain Sh41g alpha-1,6-glucosidase gene, partial cds.
