# Supplementary material for: Development of a Visual Assay for Detection of Viable Cronobacter sakazakii Using RT-PSR and Hydroxynaphthol Blue Indicator
Source: Biology (Basel). 2025 Apr 7;14(4):383. doi: 10.3390/biology14040383 (PMC12024772; doi:10.3390/biology14040383)
Supplement: Supplementary file 1 [file biology-14-00383-s001.zip › biology-3514590-supplementary.pdf]

Supporting Material

***Cronobacter sakazakii* strain Sh41g alpha-1,6-glucosidase gene, partial cds**

GenBank: JX315550.1

ORIGIN

1 agctttatgg acgccaacgg cgacggcgtg ggcgacctgg caggcatcat cagcaagctc
61 gactatctgc aacagcttgg catcaacctt atctggctct cgccggtcta caaatcgccg
121 atggacgaca atggctacga tatctccgac tacgacgata tcgccgcgga gttcggcacg
181 atggcggaga tggagcaact gattcaggaa gcgaaggcgc gcgatatcta catcctgatg
241 gatctggtgg tgaaccacac ctctgacgag catccgtggt ttctcgaggc gaagaaaggc
301 aaagataacc cgtaccgcga tttctatatc tggcgtaaac cggcgccgga cggcggcccg
361 ccgaatgatt accgctcgca ttttggcggc agcggctggg cgtatgacga ggcgagcggc
421 gaatattatc tgcaccagtt ttcggtgcgc cagccggatc tcaactggga aaacccgcgc
481 gtgcaggagg agatccacgc gatgatgaac cgctggctgg ataagggcat cggcggtttc
541 cgcatggacg ttatcgattt aatcggcaaa gaggttgacc ggcagatcat ggcgaacggc
601 aagcacctgc acgtgctgct gcgccagatg aacgaggcga cgttcggccc gcgcgattcg
661 ctgaccgtgg gcgaagcctg gagcgccacg ccggaagacg cgctgctcta cagcgatccg
721 gaacgccggg aactctcgat ggtgtttcag tttgaacata ttaaacagac ctgggatgaa
781 aaagcgggca agtggcgcag caggccgttc gagctgtcgc gctttaaagc agtgattgat
841 aagtggcaga cggcgctggc cgaccgcggc tggaactcgc tgttctggag taatcacgac
901 ctgccgcgcg cggtgtcgaa atttggcaat gacggcgagt ttcgtgaggt ctcggcgaaa
961 atgctcgcca ccgcgctcca ctgcctgcgc ggcacgccct atatctatca gggcgaggag
1021 atcggcatga ccaacgtgcg ttactccacc atcgaagagt atcgcgatat cgaaagcctt
1081 aatttctacc gggaactcat cgcaggcggc ctgacgcatg acgagatgat gaccggcatc
1141 tacgccaacg gccgcgacaa cgcccgcacg ccgatgcagt gggatgacag cccgaacggc
1201 ggcttcacga ccggcaggcc gtggctcggc gtgaatccca attaccgcga gattaacgtg
1261 gcgcaggcgc tggccgagcc tgactctatt ctctggcatt atcaaaaact ggtggcgctg
1321 cgtaaacagt acccgattct ggtgtatggc gattatcaga tgctgtttgc cgaacacccg
1381 gaagtgttcg cctgggtgcg ccgctacgag ggcgacacgc tgctggtgat aaataacttt
1441 tttggcaacg ccattacgtt gcccattccg gaggcgatgc aggcgtggca cggcgaatgc
1501 cttatcagca actatgcgcc gcgtgaccag
